# A Heterogeneous Architecture for the Vision Processing Unit with a Hybrid Deep Neural Network Accelerator

**DOI:** 10.3390/mi13020268

**Published:** 2022-02-07

**Authors:** Peng Liu, Zikai Yang, Lin Kang, Jian Wang

**Affiliations:** 1 School of Microelectronics, Tianjin University, Tianjin 300072, China; zationlue@tju.edu.cn; 2 School of Artificial Intelligence, Hebei University of Technology, Tianjin 300401, China; 2021094@hebut.edu.cn; 3 China Mobile Group Henan Co., Ltd. of Network Management Center, Zhengzhou 450008, China; kanglin@ha.chinamobile.com; 4 Qingdao Institute for Ocean Technology, Tianjin University, Qingdao 266200, China

**Keywords:** vision processing unit, deep neural network processing unit, image signal processing, hybrid deep neural network

## Abstract

The vision chip is widely used to acquire and process images. It connects the image sensor directly with the vision processing unit (VPU) to execute the vision tasks. Modern vision tasks mainly consist of image signal processing (ISP) algorithms and deep neural networks (DNNs). However, the traditional VPUs are unsuitable for the DNNs, and the DNN processing units (DNPUs) cannot process the ISP algorithms. Meanwhile, only the CNNs and the CNN-RNN frameworks are used in the vision tasks, and few DNPUs are specifically designed for this. In this paper, we propose a heterogeneous architecture for the VPU with a hybrid accelerator for the DNNs. It can process the ISP, CNNs, and hybrid DNN subtasks on one unit. Furthermore, we present a sharing scheme to multiplex the hardware resources for different subtasks. We also adopt a pipelined workflow for the vision tasks to fully use the different processing modules and achieve a high processing speed. We implement the proposed VPU on the field-programmable gate array (FPGA), and several vision tasks are tested on it. The experiment results show that our design can process the vision tasks efficiently with an average performance of 22.6 giga operations per second/W (GOPS/W).

## 1. Introduction

**Figure 1:**
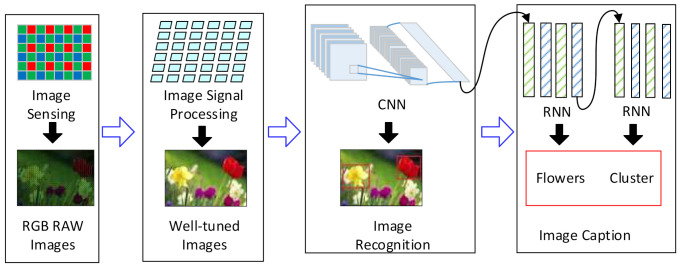
The modern vision tasks. They are composed of the ISP tasks followed by the CNNs or the hybrid DNNs.

The vision chips have shown excellent performance on the vision tasks by connecting the image sensor directly with the parallel vision processing unit (VPU) [1,2,3]. They can solve the bottlenecks of the massive image data transmission and processing in the vision tasks. They generate high-quality images and extract the required information from the images [1,4,5]. The VPU is the dominant part of the vision chip and usually utilizes the single-instruction-multiple-data (SIMD) array of processing elements (PEs). VPUs in early works [2,5] were mainly composed of the arithmetic and logic unit (ALU) array. They can accomplish the image signal processing (ISP) tasks and some computer vision algorithms like speed-up robust features (SURF) [6] at high speed. Since the neural networks have been widely used for computer vision applications, some works [1,4] have tried to reconfigure the ALU array to process the neural networks like the self-organizing map (SOM) neural networks. In recent years, deep neural networks (DNN) have proved to be very efficient and have become the most commonly-used tools for computer vision tasks [7]. For example, convolutional neural networks (CNNs) are widely used for image recognition. The hybrid neural networks [8,9] that combe CNNs and recurrent neural networks (RNNs) can also be used for some specific applications such as image caption and video description [10,11]. Generally, the modern vision tasks are usually composed of two parts: the ISP and the DNNs, including the CNNs and hybrid DNNs, as shown in Figure 1. The modern VPU should be able to process the DNNs as well as the ISP algorithms [12,13,14].

**Figure 2:**
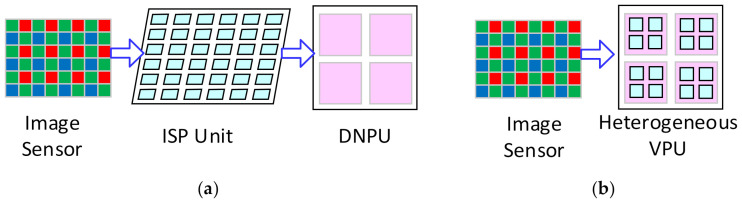
The vision systems: (**a**) The current vision systems with the ISP unit and the DNPUs; (**b**) The vision systems with the proposed heterogeneous VPU connected with the image sensor directly.

However, the traditional VPUs based on the ALU array cannot process the DNN efficiently [15]. Instead, a lot of deep neural network processing units (DNPUs) have been proposed based on the SIMD array of the multiply accumulators (MACs), and many efforts are made to shift them closer to the sensors [3,16,17,18,19,20]. However, they cannot execute the ISP algorithms [1,7], while some ISP algorithms are essential for the vision systems, including the demosaicing [14,21]. Therefore, those DNPUs cannot directly process the image data from the image sensor. Moreover, high-quality images are also required in many application areas, such as closed-circuit televisions and IP cameras [19], and the ISP algorithms are required to well-tune the images. Therefore, an extra device for the ISP tasks is required to connect the image sensor and the DNPU when it is applied in the vision system [16,18,20,22], as shown in Figure 2a. This requires more hardware resources and consumes more power for the massive image data transmission. Therefore, a VPU that can process both the ISP algorithms and the DNNs is required to solve this problem, as shown in Figure 2b.

Furthermore, the CNNs and RNNs also have very different computing flows. The CNNs mainly consist of the convolutional and full-connection (FC) layers, while the RNNs are composed of several matrix-vector multiplications and element-wise multiplication operations [8,23]. Most DNPUs are proposed for the CNNs or RNNs, respectively [7,24,25]. Some DNPUs can process both the CNN and RNN with two respective units included for each one [24,26,27]; they are efficient for the hybrid DNNs. However, a lot of vision tasks only contain the CNNs, and the RNNs are not used in them, such as image recognition and object detection. When processing those tasks, the CNN accelerator will run alone, and the RNN accelerators will be idle, resulting in the hardware’s waste. To date, only a few works [8,25] have tried to implement both the CNNs and RNNs on one unit for the hybrid DNNs. However, they have not considered the fact that the convolutional layers consume much more computation than the FC layers/RNNs. They allocate the same hardware to both the convolutional and FC layers/RNNs. Therefore, they need to process the DNNs with a large batch of many images, such as 16 images. The convolutional layers of the batch will first be processed, image after image, which will consume a large amount of time. After that, the FC layers and RNNs for the whole batch will be processed simultaneously. When processing a large batch, they can achieve a small average latency for each image. However, when processing fewer images, the latency of each image will increase greatly, and a lot of hardware will be idle during the processing of the FC layers and the RNNs. This is not practical for real-time vision tasks [28]. Therefore, none of the state-of-the-art DNPUs are suitable to process the DNNs in modern vision tasks. The CNNs and the hybrid DNNs should be processed with one unit while the hardware allocation is customized with the different requirements of the subtasks. Much more computing resources should be allocated to the convolutional layers than the others [7,8,24].

This paper attempts to implement the ISP, CNNs, and hybrid DNNs with one VPU architecture to achieve high efficiency in both hardware utilization and power consumption. We find that the ISP units and the DNPUs share many hardware requirements. Firstly, the SIMD array is the main architecture for both [7,29], so it is possible to implement them with one SIMD array. Secondly, although the MACs are the dominant components for the DNPUs, the ALUs are also essential for the non-multiplication-and-accumulation (non-Mac) operations such as pooling, activating, biasing, quantization, and batch-normalization. Besides, both the ISP units and the DNPUs require a lot of on-chip memory in their respective forms. Meanwhile, in the DNNs, the FC layers have a lot in common with the RNNs, and they can be exploited to run on the same hardware resources [7,23]. Therefore, the vision tasks can be divided into three subtasks: the ISP algorithms/non-MAC operations, the convolutional layers, and the FC layers/RNN. A sharing scheme for the subtasks can significantly improve the hardware utilization efficiency in the VPU.

Based on the requirements mentioned above, we proposed the VPU architecture with three processing modules: the ISP/non-Mac modules, the convolutional layer module, and the FC/RNN module. The modules are shared by the ISP, CNN, and RNN tasks. The VPU can process the subtasks concurrently. However, the subtasks are serial in a vision task. This means when one module is processing an image, other modules will be idle and wait, which will cause great waste of the hardware. Therefore, instead of processing the images one by one, a pipeline strategy should be applied to process several images concurrently with different subtasks. This can eliminate the idle time of the processing modules, and the VPU can achieve higher processing speed with the same amount of hardware resources.

We summarize three key points to design an efficient VPU from the analysis mentioned above. Firstly, the customized processing modules should efficiently meet the computing requirements of different subtasks. Secondly, the processing modules should be multiplexed for the different tasks to save the hardware resources. Finally, the VPU should be able to carry out the vision tasks in a pipelined way to eliminate the idle time of the processing modules. In this paper, we propose a heterogeneous architecture for the VPU that can execute both the ISP and the DNN tasks. A 2-D SIMD PE array is designed, composed of the ALU array and the MAC array. It can process the ISP tasks, the convolutional layers, and the non-Mac operations in the CNNs. The FC layers and the RNNs will be processed by a 1-D Row Processor. The hardware resources are highly multiplexed among the different tasks. The convolutional layers and the FC layers/RNNs can be processed in a parallel way with a batch of only two images for the CNNs and hybrid DNNs with negligible idle time on the hardware. A pipelined workflow is applied to process the vision tasks seamlessly. It will improve the utilization efficiency of the hardware and achieve higher power efficiency. To the best of our knowledge, this work is the first design to efficiently implement the ISP, CNN, and hybrid DNN tasks on one VPU with a pipeline strategy.

The main innovations in this work are listed as follows.

- A heterogeneous VPU architecture with a hybrid PE array and a 1-D Row Processor is designed to implement the ISP, the CNNs, and the hybrid DNNs tasks. The time-sharing schemes are applied to multiplex the hardware resources for different tasks.
- A new workflow for the DNNs is proposed with customized processing modules to process the subtasks of the DNNs concurrently.
- A pipeline strategy is applied to seamlessly carry out the vision tasks without any notable idle cycles on the processing modules.

The rest of the paper is organized as follows. Section 2 discusses the background of this work. Section 3 introduces the architecture of the proposed VPU, and Section 4 details the workflow for the vision tasks based on it. The experimental results and the discussion are presented in Section 5. Finally, the paper is concluded in Section 6.

## 2. Preliminary

Modern vision tasks consist of the ISP algorithms and DNNs. In the application areas, such as industrial automation, security monitoring, and autonomous navigation, CNNs are the most commonly-used tools in vision tasks [18,19,20,22,30]. They are used directly for image recognition or as the backbone of objection detection networks. On the other hand, hybrid DNNs are also applied for some fields within the CNN-RNN framework [31,32]. Generally, ISP and CNN tasks are required for all vision tasks, and the RNNs are also used in some cases. To achieve high power efficiency and high processing speed, the VPU should exploit the sharing scheme of the hardware resources for both the ISP algorithms and the DNNs. Moreover, the VPU should be able to execute the CNNs alone, and the hardware resources for the RNNs should be multiplexed by the CNNs.

To implement the ISP algorithms and DNNs with the same VPU, we firstly studied the difference between the ISP unit and the DNPU. The former is based on the von Neumann architecture, while the latter is based on the non-von Neumann architecture with specific computing resources, fixed data flow, and deterministic data reusability [1,7]. The ISP units utilize the ALUs with common functions, including addition, subtraction, and logical operations [1,2,4,5], while the primary computing components in the DNPUs are the MACs. Besides, the small distributed on-chip memories are applied in the ISP units for each ALU to carry out the middle-level algorithms on the neighborhood pixels [1,2,4,5,33,34], while the large-size memories are required in the DNPUs for the massive intermediate data and weights.

Secondly, we analyze the difference in the computing flow of the CNNs and the RNNs. Generally, the main computation of the DNNs comes from three classes of layers, including the convolutional layers, the FC layers, and the RNN gates [7,8]. The convolutional layers are computation-intensive. Both the input data and the weights are heavily reused in them. The FC layers are memory-intensive with a great number of weights in them. No weights are reused in the FC layers, while all the input data are shared in each layer. Besides, the convolution accounts for 90% of the computation in the CNNs [15], while the dominant computation in the RNNs is the matrix multiplication [7,8]. This results in the different hardware resources requirements for the CNNs and RNNs.

As mentioned above, the ISP unit can share the same processing module with the pooling and activating unit of the DNPU. The primary operations in the RNNs are matrix-vector multiplication and element-wise multiplication. The former operation is the same with the FC layers, while the latter is similar. Therefore, the RNNs and the FC layers can be exploited to run on one processing module, while another module with more MACs is required to process the convolutional layers. The VPU is proposed by integrating those three processing modules.

The VPU should be shifted to the image sensor as close as possible. In some works, the processing elements (Pes) of the VPU are coupled tightly with the photodiode in the image sensors to achieve the best power efficiency, but they suffer a significant loss in the fill factor and the resolution of the image sensor [2]. Therefore, designing an independent VPU and connecting it with the image sensor is a more practical approach. The independent VPU can be integrated with the sensor into one chip [4,5] or one board [1]. The VPU is recommended to work as a co-processor with the microprocessor. In this case, the VPU will process the image data directly from the sensor and send the results to the microprocessor, and the microprocessor will instruct the VPU.

Since the VPU will process the output pixels from the image sensor directly, there should be a ratio between the resolution of the sensor and the size of the 2-D SIMD PE array in the VPU. The pixels from the sensors are supposed to be evenly allocated to the Pes to make full use of the array during both the ISP and the CNN tasks. Therefore, the resolution of the image sensor should be the multiple of the size of the PE array in the VPU. On the other hand, the sizes of the initial input images of the CNNs are not required to be fixed. The images with varied dimensions can be processed by the CNNs with the pooling techniques such as spatial pyramid pooling (SPP) [35] and global pooling [36]. Consequently, various CNNs can be fully implemented on the VPU with the fixed array size.

## 3. The Architecture of the VPU

### 3.1. The Overall Architecture

**Figure 3:**
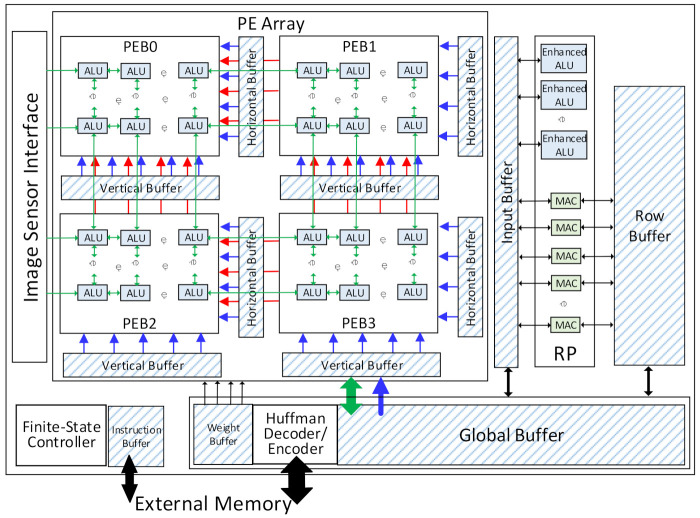
The overall architecture of the VPU.

**Figure 4:**
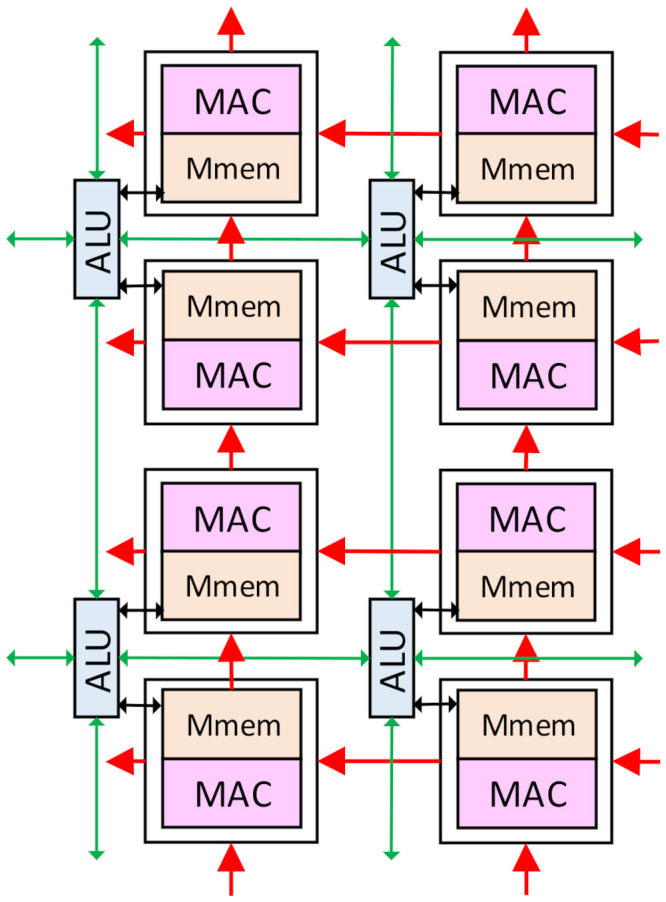
The connection of the ALUs and the MACs.

As shown in Figure 3, the VPU consists of a 2-D PE array, a row processor (RP), a global buffer, and several specific-application buffers. The 2-D PE array comprises four PE blocks (PEBs) and one ALU array. Each PEB has a vertical buffer and a horizontal buffer to provide the input data during the CNN tasks, while the weights are accessed from the weight buffer. The ALUs are embedded in the PEBs by connecting each ALU with two Pes, as shown in Figure 4. The ALUs can process the data from the Pes. The leftmost column of ALUs can also get the RGB-RAW image data from the image sensor interface directly and perform the ISP algorithms as required. The row processor is composed of a 1-D MACs array and several enhanced ALUs. It has a row buffer and a sharing buffer. The global buffer is the main on-chip memory. It stores the prefetched input data from the external memory and the intermediate data from the PE array and the row processor. The data exchange between the VPU and the external memory is accomplished through the Huffman encoder/decoder module. All the intermediate data and the weights data for the DNNs are compressed with the two-symbol Huffman coding before being stored into or loaded from the external memory. A finite-state controller (FSC) decodes the instructions and generates the control signals for each module in the VPU.

### 3.2. The PE Array

The PE array is the dominant processing module in the VPU. The convolution operations of the CNN and the ISP algorithms are executed on it. The PE array is a hybrid architecture of an ALU array and four PEBs.

The PEB is an m × m SIMD array of Pes. Each PE consists of a MAC unit, several registers, and a small distributed memory named the Mmem in this paper. The MAC units can contain one or several MACs, with each MAC working in the same manner with different data. This technique is used to improve the computing power of the PE array. The PE with one MAC will be discussed in this paper as an example, and other types can be learned by analogy. Each PE is connected with the upper, lower, right, and left neighbor Pes. The Pes can get data directly from its Mmems and process it. The data in each PE can also be transferred to the upper and left Pes as the inter-PE transmission. This enables the PE to perform the 2-D convolution for the CNNs. Each PEB has a horizontal buffer connected with the rightmost column of the Pes and a vertical buffer connected with the bottom row. The input data can be accessed from the vertical or the horizontal buffers to the connected Pes and then transferred to the upper or the left Pes. This is the transmission path that loads the input data into the Mmems in the PEBs. Those buffers will also provide the input data during the convolution computing, as illustrated in Section 4. An alignment-transmitter is used in each vertical/horizontal buffer to prepare the input data before they are transmitted to the PEB, mainly consisting of several registers. A weight buffer with four banks is connected to the PE array, with each PEB directly getting data from one bank. At each convolution computing cycle, four data from the weight buffer will be sent to each PEB. Then, each data will be broadcasted to all the Pes in one PEB. All those buffers can access the input data from the global Buffer or the Huffman encoder/decoder module.

**Figure 5:**
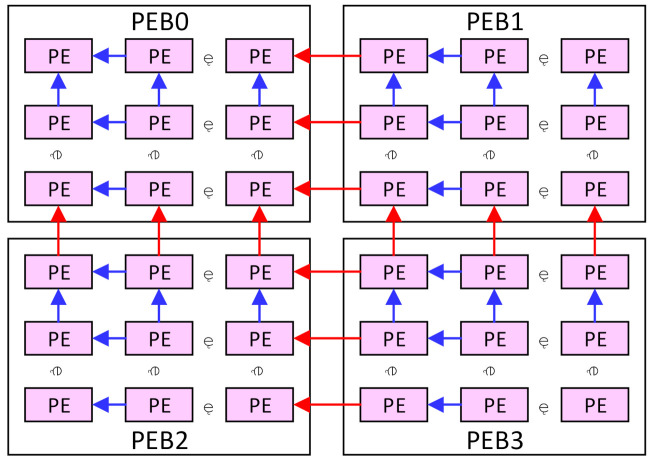
The connection of the PEBs.

As shown in Figure 5, the PEBs are also connected with each other, and the data transmission can be performed both intra and inter the PEBs. The rightmost column of the PEB0 and PEB2 can access data from the leftmost column of the PEB1 and PEB3, respectively, and the bottom row of the PEB0 and PEB1 can access data from the top row of the PEB2 and PEB3, respectively. This makes four PEBs work as a 2m × 2m array or two 2m × m arrays, as discussed in Section 4. In this case, the four banks in the weight buffer will work as one or two banks, respectively, and weights can be shared between the PEBs.

The ALU array is used for the ISP algorithms and the non-Mac operations in the CNNs, such as pooling, activating, biasing, batch-normalization, and shortcut addition. The size of the ALU array is m × 2 m, and each ALU is connected with two Pes in a column. Each ALU is also connected with the four neighbor ALUs in the upper, lower, right, and left positions. The ALUs can exchange data with the neighbor ALUs for spatial computation, which is widely used in the ISP algorithms and the pooling layers. The ALU can also exchange data with the Mmems in the connected Pes. The leftmost column ALUs will receive the image pixels from the sensor interface, and the bottom row can directly exchange data with the global buffer.

### 3.3. The Row Processor

The row processor is a 1-D array with R MACs and R/2 enhanced ALUs. Each enhanced ALU is a regular ALU with the sigmoid function units. Each MAC can get two data respectively from the row buffer and the sharing buffer at each cycle. This is very effective for element-wise multiplication. The MACs are connected one by one, and they can share input data as one of the multipliers at each cycle when processing the FC layers. The shared data, in this case, is from the sharing buffer and transmitted to the first MAC before it is broadcasted to the whole row. The MAC will send its results to the sharing buffer. The enhanced ALUs can read and process the data after that.

### 3.4. The Memory Architecture

As mentioned above, there are several different forms of on-chip memories in this VPU, including the global buffer, the specific buffers in the processing modules, and the Mmems in the Pes. The global buffer is the main module to exchange data with the external memory. It caches the intermediate data to reduce the repeated data access from the external memory and prefetches the input data for the processing modules to eliminate the memory bottleneck. Other buffers can get data from the global buffer.

The Mmems in each PE is the main module to store the input data for the convolutional layers. The MACs can access the input data from the local Mmems and transmit them to the neighbor Pes. This exploits the reusability of the input data. They also store the image pixels for the ISP tasks. The vertical and horizontal buffers are the small memories to cache the input data for the convolution computation and prefetch the input data to load into the Mmems. It can load data from the global buffers or directly from the external memory.

The row buffer mainly stores the weights for the FC layers in the CNNs and the matrix multiplication in the RNNs. It is much larger than the sharing buffer, which is used to cache the input data. The row buffer can load weights from the external memory, while the sharing buffer will access the input data from the global buffer.

All the on-chip buffers work in the double-buffering way to exploit the parallel data exchange. The vertical/horizontal buffers can directly read data from the external memory if the data is not prefetched in the global buffer and write to the external memory if the data will not be used again or the global buffer is full. It should be noticed that the Huffman decoder/encoder will accomplish all the data exchange with the external memory for the DNNs.

## 4. The Workflow of the Vision Tasks on the VPU

The top flow of the vision task is described as follows. Firstly, the ALU array should process the RGB-RAW image pixels for the ISP algorithms and transform them into fine-tuned RGB images. Then the PE array will process the RGB image as three input channels for the convolutional layers and pooling/activating layers. It will generate the feature vector of the image. At last, the feature vector will be processed by the row processor for the FC layers or RNNs. The final results will be directly sent to the external memory for further processing. The detailed flow for each subtask will be introduced in the following passages, and the pipeline strategy for the complete vision tasks will be discussed finally.

### 4.1. The Workflow for the ISP on the VPU

Each column of the image pixels from the sensors will be acquired by the leftmost column of the ALU array and then transmitted to the right ALUs column by column. After a pixel reaches the predefined ALU, it will be stored in the Mmems of the Pes connected with the ALU. The image pixels will be evenly distributed to the ALUs, and each ALU can store a tile of pixels in the Mmems. It should be noted that the size of the tiles is flexible, as illustrated in [1,33]. It is not necessary to store the whole image in the Mmems. The image can be divided into several patches. The ALU array can store one patch at one time and execute the ISP algorithms on the stored pixels. Other patches will be cached in the global buffer or even the external memory. This enables the sharing of the Mmems for the ISP tasks and the convolutional layers since the convolutional layers have the priority to use the Mmems.

The ALU can process the image pixels from the Mmems or the adjacent ALUs. It can execute both the pixel-level algorithms, including the demosaicing, and the middle-level algorithms, such as the 2-D filtering and the discrete cosine transform (DCT).

The ALU array is widely used in the early works [1,2,4,5,33] to execute the ISP algorithms. In our design, a similar computing flow is applied with the new data path mentioned above. For brevity, the detailed computing flow is not repeated in this paper.

### 4.2. The Workflow for the CNN on the VPU

The CNNs consist of the convolutional layers, pooling layers, activating layers, and the FC layers. Some irregular operations are interleaved between specific layers. The data in the CNN include the input data and the weights, which have different reusages in the convolutional layers and the FC layers.

#### 4.2.1. The Workflow for the Convolutional Layers

(1) The Mapping Scheme

The convolutional layers are processed on the four PEBs with m × m Pes in each. The input data of each layer are reused to compute every output feature map, and each kernel is reused for a corresponding input map. The input data will be cached in the vertical/horizontal buffers and the Mmems of each PEB. They will be accessed for convolution computing directly. The convolutional layers will be processed on the PE array in the modified output-stationery way. Each output map of one convolutional layer will be computed by the PE array one by one, and each PE will be dedicated to the computation of one output data at a time. Each output map will be segmented into one or several patches, and the PE array will process an output map patch by patch. To achieve the high utilization of the Pes, the output maps should be mapped on the PE array with the least idle Pes, despite the different dimensions of the output maps among the CNNs and layers. Therefore, a new scheme to efficiently map a convolutional layer on the PE array is proposed here, as illustrated below.

The PE array can work in the 2m × 2m, 2m × m, and m × m modes, as mentioned in Section 3, and each mode can process the output patches with close dimensions. For example, assume the size of an output map is L × L. L can be expressed as below:

L = 2m × p + m × q + b(1)

where p is a non-negative integer, q is 0 or 1, and b is a non-negative integer smaller than m. Then each output map can be segmented as several patches of size 2m × 2m, 2m × (m + b), (m + b) × (m + b), 2m × m, 2m × b, m × m, m × b, and b × b. We will map the patches of size 2m × 2m, (m + b) × (m + b) and 2m × (m + b) on the PE array with the 2m × 2m mode, the patches of size 2m × m and 2m × b with the 2m × m mode, and the patches of size m × m, m × b, and b × b with the m × m mode, assuming the b is not 0. The mapping scheme for each mode is detailed as below.

**Figure 6:**
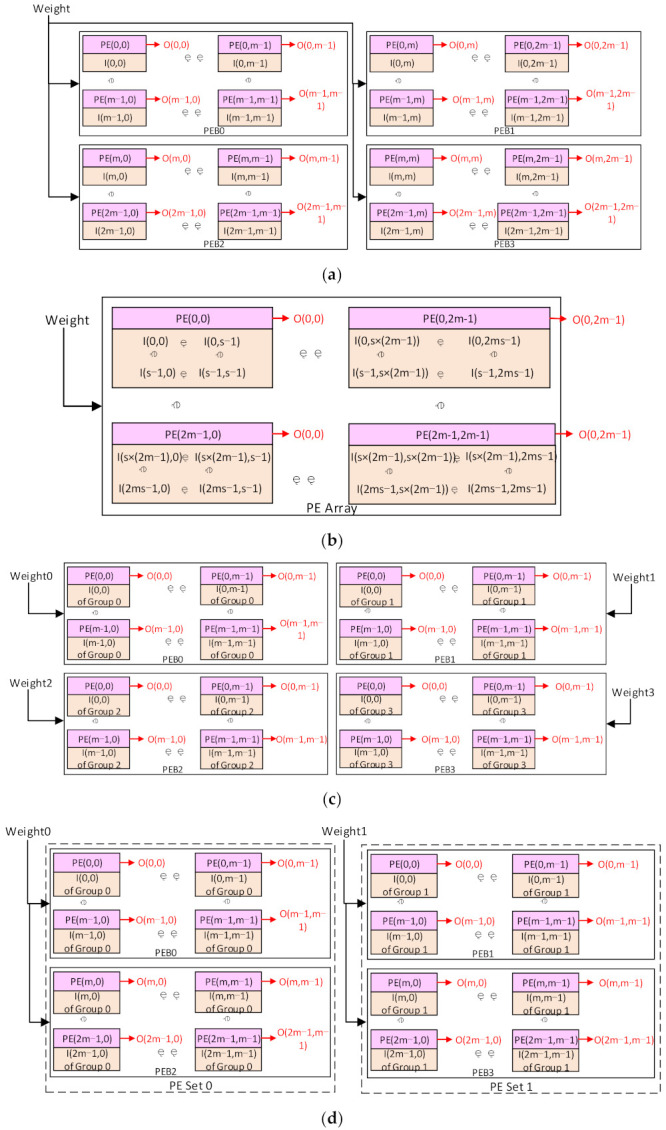
The mapping scheme: (**a**) The 2m × 2m mode with the kernel stride of 1. (**b**) The 2m × 2m mode with the kernel stride larger than 1. (**c**) The m × m mode with the stride of 1. (**d**) The 2m × m mode with the stride of 1.

First, the 2m × 2m mode with the kernel stride of 1 is considered. Each PE in the 2m × 2m array will compute the output data with the same location in the 2m × 2m output patch. It also stores the input data with the same location in the input patch. The location here denotes the relative position in the patch and the PE array. Take the output patch of 2m × 2m as an example, assuming the convolutional layers have I input channels and O output channels with the kernel size of k × k. An input patch of (2m + k – 1)^2^ in each input map is required to compute an output patch, which forms an input block of I patches. Assume the PE(x,y), I(x,y), and O(x,y) denote the PE, the input data, and the output data at row x and column y in the PE array and the input/output patch, respectively. Then O(x,y) will be computed by the PE(x,y), and then I(x,y) will be stored in the Mmems of the PE(x,y), as illustrated in Figure 6a. Each Mmem will store I input data, and the PE array will store I × 2m × 2m input data with all the Mmems. A shared weight will be broadcasted to every Pes and computed with the different input data at each cycle. Each output patch will be obtained by computing the input block with the corresponding kernels. The input block will be reused for the computing of O output patches. After the computation for an output block, a new input block will be loaded into the PE array for the new output block.

It can be noted that only 2m × 2m data of each input patch can be stored in the PE array. The rest data of the input patch will be cached in the horizontal buffers of the PEB1 and PEB3 and the vertical buffers of the PEB2 and PEB3. Specifically, each horizontal buffer of the PEB1 and PEB3 and the vertical buffer of the PEB2 will store m × (k – 1) input data, while the vertical buffer of the PEB3 will store (m + k – 1) × (k – 1) input data. Once those data are stored in those buffers, they will be computed for at least k × k cycles. It is not necessary to cache the rest data of all the input patches in the vertical/horizontal buffers. They can be prefetched and replaced during the k × k computing cycles. This can significantly reduce the capacity requirement for those buffers. If the output patches are of size 2m × (m + b) or (m + b) × (m + b), there will be idle rows and columns of Pes. The Mmems of the idle Pes will also be used to store the rest data with the same location. In this case, the rest data of all the input patches will be stored in the corresponding Pes if the Mmems are large enough. This scheme will also be used for other modes.

Second, if the stride is larger than 1 in the 2m × 2m mode, denote the stride as s, and the size of each input patch will be (2m × s + k – s)^2^, as shown in Figure 6b. Each output data will still be computed by the PE with the same location. However, the input I(0,0) to the I(0,s – 1) in the input patch will be computed for the output O(0,0), but not be used for the O(0,1) to O(0,s – 1). This suggests that it is meaningless to store the input I(0,1) to I(0,s – 1) in the Mmems of the PE(0,1) to PE(0,s – 1). Therefore, they will all be stored in the Mmems of the PE(0,0). It can be deduced that a tile of s × s input data I(0,0) to I(s – 1,s – 1) will all be stored in the Mmems of the PE(0,0). This can be generalized to any PE(x,y). The tile of s × s input data I(sx,sy) to I(sx + s – 1,sy + s – 1) will be stored in the PE(x,y), and the output data O(x,y) will be computed here. Finally, each Mmems will store I × s × s input data, and the PE array will store an input block of I × (2m × s)^2^. The rest of the input patch will still be stored in the vertical/horizontal buffers.

Third, for the m × m mode with the kernel stride of 1, since the output patch is smaller than or equal to m × m, it can be mapped on only one PEB in the traditional output-stationery way. This will make the other PEBs stay idle, and it is not efficient in the utilization of the hardware. Therefore, in this paper, we map each output patch on four PEBs, as shown in Figure 6c. Take the output patch of m × m with the kernel k × k as an example. To compute this output patch, an input block of I input patches is required. The size of each input patch is (m + k – 1)^2^, and each input patch will be stored in a PEB. Instead of storing the input block in one PEB, we divide the I input patches into four groups evenly. Each group has I/4 input patches and will be stored in one PEB. Each input data in the input patch will be stored in the PE with the same location in the PEB. For each group, m × m × I/4 input data will be stored in the Mmems of one PEB, and the rest data in the input patches will be cached in the horizontal and vertical buffers of the PEB. Each vertical buffer will cache (k – 1) × (m + k – 1) data of each input patch, while each horizontal buffer will cache (k – 1) × m data. The PE will also compute each output data with the same location in the PEB. Four different weights will be broadcasted in four PEBs at each cycle. Each PEB can compute the convolution on a group of input patches stored in it and get the partial results of the output patch. Those partial results will be sent to the ALUs and transferred to the ALU array in the PEB3. Then they will be added to generate the complete output patch of m × m data.

This scheme can be generalized to any kernel stride in the m × m mode. With a kernel stride larger than one, the size of the input patches will be (m × s + k – s)^2^. The input patches will still be divided into four groups, and each group will be stored in one PEB. Each PE will store an s × s tile of the input patch; s × s × I/4 input data will be stored in each PE, and each PEB will store (m × s)^2^ × I/4 input data in the Mmems. Then, the partial results can be produced and added to generate the complete output patch.

Finally, for the 2m × m PE array mode, four PEBs will be divided into two vertical PE sets, as shown in Figure 6d. PEB0 and PEB2 are in one set, and the other two PEBs are in another set. Each set works as a 2m × m PE array. The input block will be divided into two groups and stored in each PE set, similar to the m × m mode. Each output data will be computed by the PE with the same location in the 2m × m PE set. At each cycle, two different weights will be broadcasted in the two PE sets, respectively. Then the two PE sets will compute and produce the partial results of the output patches. At last, the partial results will be transferred to the right PE set and added to generate the complete output patch. This scheme can be generalized to any kernel stride with the analogy from the other modes.

It should be noted that in the 2m × 2m mode, only four vertical/horizontal buffers are used to cache the rest data, while the 2m × m mode uses six buffers and the m × m mode uses all eight buffers. Since the inter-PE data transmission is more energy-efficient than the buffer access, more buffers usage will consume more power. Therefore, when segmenting an output map into multiple patches, the patches for the 2m × 2m mode should obtain as many as possible, and the m × m mode should be the least used.

As mentioned above, a lot of input data will be stored in the Mmems. There may be a case that the Mmems are not large enough to store the required input data. Assume the capacity of the Mmems is C, and the number of the input data required to be stored in each Mmem is N. If N > C, the N input data will be divided into N/C groups. Each group contains less than C input data. Each Mmem can store a group of input data. Consequently, 2m × 2m groups of input data will be stored in the PE array at one time. Then the PE array can compute them and produce the partial results of the output patches. The partial results will be stored in the global buffer or the external memory. After all the output patches have been computed on the 2m × 2m groups of input data, those groups will be replaced by other 2m × 2m groups. Then, the computation will continue and produce the new partial results. After all the N/C groups are computed, all the partial results will be added together by the ALUs to generate the complete output data. It should be noticed that this case will rarely happen if the Mmems are large enough compared with the number of the input data. Our research shows that Mmems with a capacity of 512 Bytes are large enough for more than 81% convolutional layers with the 8-bit input data, and the 1-KB Mmems are practically sufficient for all the popular CNNs.

(2) The Computing Flow

Based on the mapping scheme of the convolutional layers, the new computing flows are proposed for the three modes. We will detail the computing flow for the 2m × 2m mode and then obtain the others by analogy.

For an output patch of 2m × 2m data, the convolution computation will be accomplished by the 2m × 2m PE array with the following steps. Assume the kernel size is k × k with a stride of 1.

**Figure 7:**
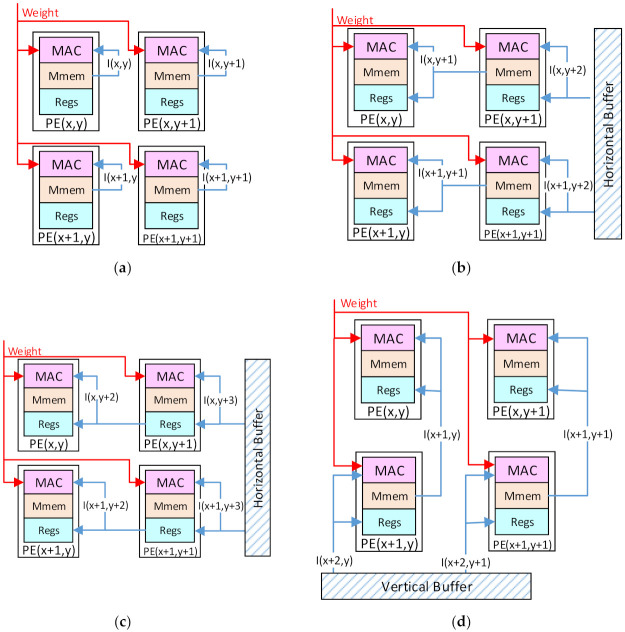
The computing flow: (**a**) Step 1; (**b**) Step 2; (**c**) Step 3; (**d**) Step 5.

Step 1: Each PE(x,y) will read the data I(x,y) of the first input patch from the local Mmems and compute it with the shared corresponding weight, as shown in Figure 7a.

Step 2: Each PE(x,y) will read the input data I(x,y + 1) from the Mmems of the right neighbor PE(x,y + 1). For the rightmost column of Pes in the array, they have no right neighbor Pes, and they will read from the horizontal buffers of the PEB1 and PEB3. Then, the input data will be cached in the registers and computed with another weight, as shown in Figure 7b.

Step 3: Each PE(x,y) continues to read the input data I(x,y + 2) cached in the registers of the PE(x,y + 1) and compute it, as shown in Figure 7c.

Step 4: Repeat Step 3 until the input data I(x,y + k – 1) is obtained and computed by the PE(x,y). So far, the convolution with the first row of the kernel is accomplished in k cycles.

Step 5: Each PE(x,y) will read the input data I(x + 1,y) from the lower neighbor PE(x + 1,y) to compute. The bottom row of Pes will read from the vertical buffers of PEB2 and PEB3, as shown in Figure 7d.

Step 6: Repeat Steps 2, 3, and 4 to accomplish the convolution with the second row in the kernel.

Step 7: Repeat Steps 5 and 6 until the convolution with the kth row in the kernel is accomplished. So far, the convolution computation with a k × k kernel on the first input patch is accomplished in k2 cycles.

Step 8: Repeat the above steps on the other input patches and accumulate the results in each PE until all the input patches are computed. Then, each PE will obtain one output data.

After the above steps, an output patch is produced, and the PE array will repeat those steps to generate another output patch with corresponding weights. The above computing flow is suitable for the convolutional layers with any kernel size. For the point-wise convolution with the kernel size of 1 × 1, only Steps 1 and 8 will be executed on all the input patches.

This computing flow can be easily generalized to any kernel stride according to the mapping scheme. The only difference is that PE(x,y) will get the input data I(sx,sy) to I(sx + k – 1,sy + k – 1) from the local Mmems. Other input data will still be provided by the neighbor Pes.

The computing flow for the mode m × m is similar to the mode 2m × 2m with a few modifications. The rightmost column of each PEB will read from its horizontal buffers as the neighbor Pes, and the bottom row of each PEB will read from its vertical buffers. After the convolution is accomplished, the partial results will be transmitted to the PEB3 and added to generate the final output patch.

The computing flow for the mode 2m × m can be deduced from the other two modes. The bottom row of each PE set will get data from vertical buffers of the PEB2 and PEB3 as the neighbor Pes. The rightmost columns of the left PE set will get data from horizontal buffers of the PEB0 and PEB2, while the other PE set gets data from the horizontal buffers of the PEB1 and PEB3. The partial results will be transmitted to the PEB1 and PEB3 to generate the final output patch.

The zero-skipping technique is applied during the computing flow. The weight buffer controller will check the weights before broadcasting to the PEBs. If the weight is zero, the skipping signal will be sent to the PEBs, and the PEBs will skip all the computations on this weight. This technique can reduce the power consumption significantly without any hardware overhead.

(3) The Data Transmission Scheme in the Convolutional Layers

The input data transmission for the convolutional layers is used to load the input data into the PE array and transfer the input data for the 2-D convolution. It includes the data access from the vertical/horizontal buffers to the corresponding rows/columns and the inter-PE transmission between each PE. In the latter one, the Pes transfer the data to the upper or left Pes.

As mentioned above, the input blocks will be stored in the Mmems of the PEBs. Before the PE array performs the convolution, the input data should be loaded into the PEBs. At each cycle, each vertical buffer can transmit m input data to the bottom row of the PEB. Then the input data will be transmitted to the upper rows at each cycle until the input data reach the scheduled PE and stored in the Mmems. It will take m cycles to load one 2m × 2m input patch, two 2m × m input patches, or four m × m input patches into the PE array through the vertical transmission path. The same data transmission flow is also performed in the horizontal direction. Input data can be accessed from the horizontal buffers to the rightmost Pes and transmitted to the left Pes. Each input patch can be loaded into the PE array by either the horizontal or the vertical transmission path.

The data transmission path is also used for 2-D convolution computing. Therefore, the data transmission path will be shared for convolution computing and data loading. We will carry out the data loading and the convolution computing in a pipelined way to achieve this. We analyze the data flow of the convolution computing. In Step 1, no data transmission is required for the computing. In Steps 2–4, only the horizontal transmission is used for the computing, while the vertical transmission path is idle. On the contrary, in Step 5, only the vertical transmission is used for the computing. It can be concluded that in each k^2^ cycles of the convolution computing with a k × k kernel, the horizontal and vertical transmission will be used for (k – 1) × k cycles and k – 1 cycles, respectively. Consequently, the horizontal transmission path can be used to load data for k cycles, and the vertical transmission path can be used for (k – 1) × k + 1 cycles. The convolution computing and the data loading will use the data transmission path alternately in a pipelined way in each k^2^ cycles.

**Figure 8:**
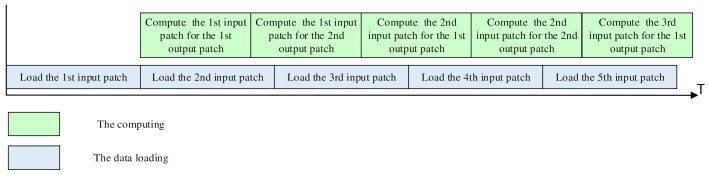
The parallel data loading and computing.

Overall, in each k^2^ cycles, (k^2^ + 1) × 4m input data can be loaded into the PE array through both the vertical and horizontal transmission paths. At the same time, the PE array requires 4m^2^ input data to compute the convolution, including a 2m × 2m input patch, or four m × m input patches. If the (k^2^ + 1) × 4m is smaller than 4m^2^, it means the data loading cannot catch up with the convolution computing. This will result in the idle state of the Pes waiting for the data loading. In this case, we will compute the input patches for more than one output patch, as shown in Figure 8. After an input patch is loaded, the PE array will compute them for the first output patch with k^2^ cycles and store the partial results in each PE. If the loading of the new input patch is not finished by then, the PE array will continue to compute the second output patch on the loaded input patch. This operation can be repeated until the new input patch is loaded. This scheme is effective for kernels of any size, including the point-wise convolution.

Moreover, the PE array can preload the new input blocks during the computing on the old input block already stored in the PE array. Assume the capacity of the Mmem is C, and D input data of the old input block is stored in each Mmem. During the computing on the old input block, (C-D) data of the new input block can be preloaded into each Mmem. When computing the old input block for the last output patch, the new input patches can directly replace the old ones in the Mmems. Therefore, when the PE array starts to compute on the new input block, some input patches have already been preloaded. While the PE array is computing the loaded input patches, the loading of other input patches will continue. Especially for the depth-wise convolution, the PE array can compute each input patch immediately after it is loaded, even though the computing on the old input block is not finished. By this means, the data loading will not bring about any delay for the computing. The PE array can process the convolution layers seamlessly without any idle cycles. Furthermore, this technique does not require a large bandwidth between the PE array and the global buffers, and the large amounts of buses connect the Pes, and the buffers are also eliminated.

When the vertical transmission is used for loading the data into the PEBs, all the vertical buffers will transmit the data synchronously, as will the horizontal buffers. The same data loading flow is applied for all three modes. The vertical and horizontal transmission will load the different input patches, respectively. The loading order of the input patches is determined by the data loading ability of the vertical and horizontal transmission.

The output data from each PE will be sent to the connected ALUs for the non-Mac operations. After the final output data are obtained, they will be transferred to the global buffer by the ALU array in the vertical direction, or directly stored in the Mmems if they will be used for the next convolutional layers and there are free spaces in the Mmems.

#### 4.2.2. The Workflow of the FC Layers

**Figure 9:**
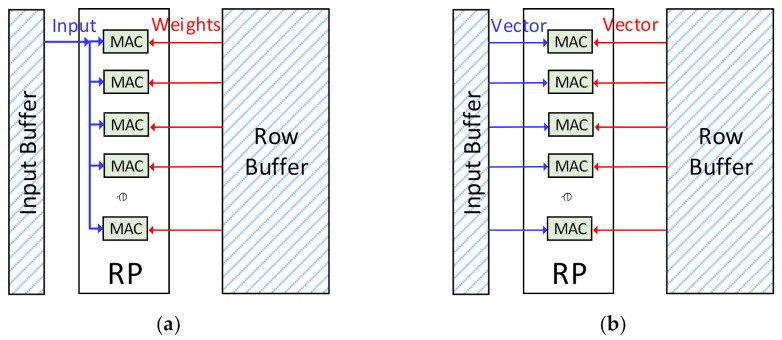
The workflow on the row processor: (**a**) The workflow of the FC layers. (**b**) The workflow of the element-wise convolution.

Different from the convolutional layers, the weights are not shared in the FC layers, while the input data are reused to compute all the output data in one layer. The FC layers are processed on the row processor, as shown in Figure 9a. The weights are stored in the row buffer while the input data is stored in the sharing buffer. The FC layers are also processed in the output-stationery way. Each MAC will compute one output data, and R output data can be computed concurrently.

When computing an FC layer containing F output data, it will be divided into ⌈F/R⌉ groups. The row processor will compute the FC layers group by group. At each cycle, one input data is read from the sharing buffer to the first MAC in the row processor and broadcasted to all other MACs. In the meantime, R weights will be read from the row buffer to R MACs and computed with the shared input data. After the computation of a group is finished, the R MACs will continue to compute the next group until the whole layer is accomplished. The results of the MACs will be sent to the sharing buffer, and the enhanced ALUs will compute them for the activating or quantization. The final output data will be stored in the sharing buffer for the next computation.

All the full-connection operations of the CNNs will be processed on the row processor, such as the squeeze-and-excitation. They will all be computed like the FC layers. The input data of them are all from the convolutional layers. The zero-skipping scheme is also applied for the FC layers. The first MAC in the row processor will check the input data and will send the idle signal to all the MACs when the input data are zero.

#### 4.2.3. The Non-Multiplication-and-Accumulation (Non-Mac) Operations

The non-Mac operations include the pooling, activating, biasing, and batch-normalization (BN) and are processed by the ALUs. For the activating layers after the convolutional layers, the results of the convolution layers will be stored in the Mmems first. Then, the ALUs connected with the PEs will read the results and compute them for the activating operations, including the Rectified linear unit (ReLU). A similar scheme is applied to activate the FC layers with the enhanced ALUs in the row processor. The BN operations will also be accomplished in this way.

Although the sigmoid function can also be processed on the ALU, it will take much more cycles than the ReLU. Therefore, for the activating layers with sigmoid after the convolutional layers, the convolution results will be transmitted to the row buffer and computed for the sigmoid function by the enhanced ALUs.

The ALU array will also process the pooling layers. Each ALU can get data from the Mmems and transmit them to the adjacent ALUs. The pooling operation with various sizes of windows can be accomplished.

The ALU array will also accomplish other non-Mac operations, such as the addition of the shortcut connection. The former input map will be reloaded into the PE array and added with the current output data to generate the final output map.

It should be noted that the non-Mac operations require much less computation than the convolutional layers or FC layers. Although fewer ALUs are contained in the VPU than the MACs, the non-Mac operations still take much less time than the convolutional layers and FC layers.

### 4.3. The Workflow of the RNN

The RNN mainly consists of matrix-vector multiplication (MVM), element-wise multiplication (EWM), and activating operations. The MVM is similar to the FC layers, and the same workflow mentioned above is applied to it. The EWM is multiplying two matrixes, A and B, to generate the matrix C with the following equation:

(2) $$c_{\mathrm{ij}}={\;a}_{\mathrm{ij}}\times b_{\mathrm{ij}}.{\;a}_{\mathrm{ij}},b_{\mathrm{ij}},c_{\mathrm{ij}}\in A,\;B,\;C.$$

It is different from the FC layers or the MVM operations. There are no weights in it, and each input vector is multiplied one or several times. When processed on the row processor, the two input vectors of the EWM will be stored in the row buffer and the sharing buffer, respectively, as shown in Figure 9b. At each cycle, each MAC will get two corresponding data of the two input vectors from the buffers. Then the two data will be computed. Each MAC will also be sent to the buffers, and the enhanced ALUs will compute them for the activating operations.

It should be noted that the tanh-function is also used in the RNNs for activating. It requires many hardware resources to implement. Considering the tanh-function is not often used in hybrid DNNs, it is not economical to equip the VPU with the specific units for the tanh function. Instead, the tanh-function can be accomplished with the sigmoid unit by the following equation, which is also applied in [23].

tanh(x) = 2 × sigmoid(2x) − 1(3)

Therefore, the tanh-function can share the enhanced ALUs, and no specific hardware units for it are necessary. The activating functions will run independently on the enhanced ALUs with the MVM and EWM operations on the 1-D MAC array. Since it takes hundreds to thousands of cycles to generate output data during the MVM operations, the runtime of the activating functions will be masked in the MVM operations.

When processing a complete RNN with several operations, including the MVM and the EWM, each operation will be executed one by one. The input and output data of each operation are usually presented in the form of vectors. The output vectors of the former operation can be cached and used as the input vectors for the latter operation. The input vectors of the MVM will always be stored in the sharing buffer, and the two input vectors of the EWM must be stored in the row buffer and the sharing buffer, respectively.

### 4.4. The Pipeline Strategy in the Workflow of the Vision Task

As mentioned above, the ISP and the non-Mac layers subtasks will be processed by the ALU array, and the convolutional layers will be processed by the PEBs, while the FC layers and the RNNs will be processed by the row processors.

The serial workflow of the vision tasks is listed as follows. The ISP subtasks are finished by the ALU array first and generate the input images. Then the convolutional layers will be processed on the images by the PEBs, while the non-Mac layers are executed on the ALU array. After all the convolutional layers and non-Mac layers are accomplished, the output feature maps or vectors will be sent to the sharing buffer, and the FC layers will be performed by the row processor. The output vectors of the FC layers will be sent to the external memory as the results of the vision tasks if no RNNs are required. Otherwise, they will be stored in the sharing buffer, and the operations of the RNNs will be executed sequentially. At last, the output of the RNN will be sent to the external memory as the final result of the vision task.

**Figure 10:**
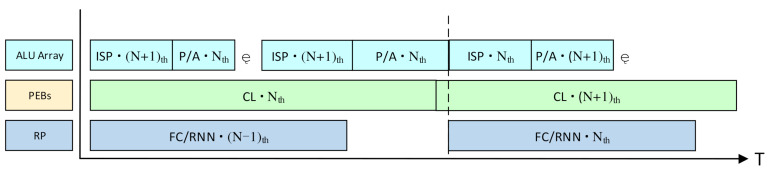
The pipelined workflow for the vision tasks. The P/A means the non-Mac operations such as the pooling and activating. CL is short for the convolutional layers. “N_th_“ means the operations for the N_th_ image.

If the VPU processes each vision task alone in a serial way, only one processing module will be working at one time, and the other modules will be idle. This will result in a great loss of hardware utilization and processing speed. Therefore, a pipeline strategy is applied in the workflow for the vision tasks to fully use the hardware resources and process three images simultaneously, as shown in Figure 10.

When the PEBs are processing the convolutional layers of the N_th_ image, the ALU array will process the non-Mac layers, such as the activating and pooling layers. Since the non-Mac layers only consume much less time than the convolutional layers, there will be a lot of idle cycles for the ALU array during the CNN processing. Therefore, the ISP algorithms for the (N + 1)th image can be executed on the ALU array in those idle cycles. Meanwhile, the row processor can process the FC layers and the RNNs on the (N − 1)th image. If the row processor has not accomplished the FC layers/RNN of the (N − 1)th image when the PE array has finished the convolutional layers for the N_th_ image, the output of the convolutional layers will be cached in the sharing buffer, and the PE array will continue to process the convolutional layers for the (N + 1)th image. The pipeline strategy is also effective for the vision tasks containing the CNNs only.

By this means, the VPU can process three images simultaneously, and the vision tasks can be executed seamlessly. The four PEBs will work consecutively without any idle cycles. Although there may be a few idle cycles for the row processor, it will be negligible for the overall hardware utilization because the row processor contains much fewer MACs than the PEBs.

## 5. The Experiment Results and the Discussion

To validate the efficiency of the proposed VPU architecture, we implement it on the FPGA, and various vision tasks are tested on it. Then the experimental results will be compared with other works and discussed.

### 5.1. The Implementation

We implemented the VPU on the test board Genesys2 with the XC7K325T-2FFG900C FPGA. The FPGA device provides the 18 × 25 DSP modules and the dual-port 36 Kb Block RAM (BRAM). The DSP modules can work as two 8 × 8 MACs or one 16 × 16 MAC. The port-width of the BRAM is up to 72 bits. The DSP forms all the MACs in the VPU, and all the on-chip buffers are comprised of the BRAMs.

Before being implemented on the FPGA, the concrete characters of the architecture were determined, including the dimension of the PE array and the row processor, the quantity of the MACs in each PE, the capacity of the Mmems, and buffers, and the bit-width of all the components.

The dimension of the PE array is determined by the resolution of the image sensor, as illustrated in Section 2. We used the 224 × 224 image sensor for the convenience of the popular CNN testbenches. Therefore, we implemented the PE array with four 7 × 7 PEBs and one 7 × 14 ALU array. There were 7 rows and 14 columns of ALUs in the ALU array. The number of MACs in the row processor was 8. Other characters in the instantiation are listed as below:

1. Each MAC in the PE array and the Row Processor was instantiated by one DSP and could work as one 16 × 16 MAC or two 8 × 8 MACs;
2. The bit-widths of both the ALUs and the enhanced ALUs were 8, and the buses were also 8-bit wide;
3. The bit-widths of the inter-PE transmission buses and the weight bus were 16 in the PE array, in which one 16-bit data or two 8-bit data could be transferred;
4. Each horizontal/vertical buffer in the PEBs was composed of 7 2-KB banks, and each bank in the weight buffer was also 2-KB;
5. The sharing buffer was comprised of 2 4-KB banks, while the row buffer consisted of 8 4-KB banks;
6. The Global Buffer is comprised of 10 4-KB banks, and each Mmem is a 1-KB RAM.

The VPU was designed with the Verilog HDL and synthesized by the design tools Vivado 2019.2. The power consumption evaluation for different testbenches was also accomplished on the simulator of the tools. The FPGA resources utilization of the VPU is shown in Table 1. Since the DNPU accounts for the most resources of the proposed VPU, it is meaningless to compare it with the traditional VPUs that did not contain the DNPU. Instead, the state-of-the-art works of the DNPUs are listed in Table 1 for comparison.

### 5.2. The Experiment Method

#### 5.2.1. The Modeling of the 224 × 224 Image Sensor

We used the color camera module PCAM 5C to model the image sensor. The PCAM 5C can provide the RGB image flow in the format of RAW10. With this camera module, we could get consecutive RGB-RAW images. Then a tile of 224 × 224 pixels was split out from each image with the same location and stored in the onboard flash sequentially. During the tests of different tasks, those tiles were sent to the FPGA as the RGB-RAW image sensor signals. This model could achieve a 224 × 224 RGB-RAW image flow at a speed of more than 1000 fps.

#### 5.2.2. The Quantization

When processing the DNNs on the DNPUs, the input data and the weights needed to be quantized to fix-point numbers. Although the quantization with high precision could maintain the accuracy of the DNNs, it would consume much more power and hardware resources. On the other side, the lower bit-width data format has shown much higher efficiency in the power and hardware. The precision of the data is the only factor that affects the accuracy of the DNNs when the DNNs are processed on the DNPUs. Therefore, previous works [7,8,23,44,45] have thoroughly studied the relation between the accuracy and precision of the DNNs, and some proper quantization schemes are proposed in those works. For the CNNs, the 8-bit fix-point quantization for both the weights and the input data has proved to be efficient and brought about negligible loss in the accuracy [7,8,28,38], as shown in Table 2.

It can be concluded that the 8-bit quantization is precise enough for the CNNs. For the RNNs, the 16-bit quantization is applied for most of the previous works [7,8,25,26,27,42], and it has achieved comparable performance with higher precision. Therefore, in our work, the 8-bit fix-point was used for the input data and weights of the CNNs, and the 16-bit quantization was used for the RNNs with the static quantization methods of the work [46].

It should be noted that the quantization scheme is not among the research points in our work since it has already been thoroughly studied. Moreover, the MACs in the proposed architecture can be implemented with any bit-width. In this experiment, the MAC in each PE was instantiated by the 18 × 25 DSP. When working as two 8 × 8 MACs, each PE got two 8-bit weights and two 8-bit inputs in each cycle. The PE can also work as one 16 × 16 MAC, and one 16-bit weight and one 16-bit input data were accessed for it. The same scheme was applied to the MACs in the row processor for the FC layers. Therefore, our design can also process the CNNs with 16-bit precision. For the RNNs with the bit-width of 16, the DSPs in the row processor will work as one 16 × 16 MAC. Technically, the computation for the DNNs with the higher precision can also be accomplished by the 16-bit MACs with more cycles, such as the 32-bit fix-point, but it is seldom used in the current DNPUs.

#### 5.2.3. The Testbench

To validate the performance of the proposed VPU, two classes of the vision tasks are tested on it. The first one is the vision tasks comprised of the ISP algorithms and the CNNs, while the RNNs are added to the other one in the hybrid DNNs. Since this work is the first design that implements the ISP unit and the DNPU within one architecture, we first ran the ISP subtasks alone and compared them with other works to evaluate the ISP unit of the proposed VPU. Then various CNNs and hybrid DNNs were processed on the VPU respectively to test the performance of the DNPU. At last, the complete vision tasks were executed on the VPU to validate the efficiency of the VPU and the pipeline strategy. The performance for the complete vision tasks was compared with the sum of the separate performance for the subtasks.

### 5.3. The Experiment Results and the Analysis

#### 5.3.1. The Experiment Results for the ISP and the Analysis

We ran some common and essential ISP algorithms on the VPU, including the demosaicing, the discrete cosine transform (DCT), and the median filter. The experiment results and the comparison with early works of the VPU are shown in Table 3. The sensors used in the early works are monochrome and can provide images with only one color, while our work adopts the RGB image sensor. This means that our design processes three channels of each image, while the VPUs in other works only process one channel. When the proposed VPU processes the ISP algorithms alone, the MACs in the PEBs and the row processor are idle.

As shown in Table 3, our VPU can execute the ISP algorithms at a high speed of more than 500 fps. The performance of the VPU for the ISP tasks is in proportion to the frequency and the size of the PE array. Although the smaller size of the PE array was adopted in our implementation, it still achieved relatively high performance of 19.6 GOPS (giga operations per second). The power consumption was higher than other works because many extra hardware resources for the DNPU were contained in our design. However, the extra hardware resources, including the larger distributed memories, improved the processing speed by caching more pixels for each ALU.

It should be noted that the DNNs are the dominant parts in the vision tasks, and it is efficient to allocate more resources to the DNPUs than the ISP units. In the meantime, the execution of the ISP algorithms should not delay the DNN subtasks in the workflow for the complete vision tasks. The experiment results show that most ISP algorithms consume less than 2 milliseconds. It is much less than the runtime of the DNNs, which usually take several to hundreds of milliseconds. Therefore, the ALU array can carry out the ISP algorithms without adding any extra overhead in the runtime of the DNNs.

#### 5.3.2. The Experiment Results for the DNNs and the Analysis

Two types of DNNs were tested on the VPU for the vision application, including CNNs and the hybrid DNNs. The CNNs were used in both types and accounted for the dominant computation. Therefore, the processing performance of the CNNs was the primary character to evaluate the DNPUs. We first test the CNNs alone to validate the efficiency of the proposed VPU. The hybrid DNNs were then processed on the VPU to verify the pipelined workflow. During the tests of the DNNs, the ISP tasks were not executed on the VPU, and all modules were used to process the DNNs.

(1) The Test of the CNNs

Firstly, the CNNs were tested on the VPU alone. To validate the applicability of the DNPUs, various CNNs with irregular operations were tested, including the VGG16, the DenseNet, and the MobileNetV2/V3-L.

As illustrated in the early works, the number of MACs and the operating frequency were the basic factors that determined the peak computing performance of the DNPUs. However, each architecture can achieve higher performance if implemented with more MACs or operating at a higher frequency. Therefore, the figures that normalize the performance with the numbers of MACs and the operating frequencies were applied to compare different architectures. Since the MACs were implemented by the DSP in the FPGA platform, the performance of the VPU was evaluated based on the DSPs. The computing efficiency of the DSP in each architecture was calculated as follow:

(4) $$\mathrm{GOPS}/\mathrm{DSP}/f=\frac{\mathrm{The}\;\mathrm{effective}\;\mathrm{GOPS}\;\mathrm{for}\;\mathrm{the}\;\mathrm{CNN}}{\mathrm{The}\;\mathrm{number}\;\mathrm{of}\;\mathrm{the}\;\mathrm{DSP}\;\times \;\mathrm{the}\;\mathrm{operating}\;\mathrm{frequency}}$$

It is shown as GOPS/DSP/f. This figure indicates the effective computing performance that each DNPU can achieve when processing various CNNs. The operations counted in the GOPS are based on the 8-bit data, and the 16-bit operation will be counted as two operations on the DSP. The effective GOPS was calculated as dividing the number of the multiplication-and-accumulation operations included in each CNN by the runtime of each CNN. The DNPUs with the higher GOPS/DSP/f can achieve performance with fewer hardware resources and power consumption. For the power estimation, the GOPS/W is the common figure to imply the power efficiency of the DNPUs. The test results are shown in Table 4.

As shown in Table 4, our design achieved a higher computing efficiency of the DSPs than most other works. The work [37] shows a better GOPS/DSP/f by considering the sparsity. It skips the zero-operations in both the weights and the input data, but it can only be used to accelerate the common convolutional layers. Its applicability is heavily limited. For example, the FC layers are not supported by it, as well as some irregular operations such as the depth-wise convolution. The work [40] also shows a high efficiency on the VGG16, but it adopts the data with mixed bit-widths as low as 2-bit. This requires fewer computational resources than the 8-bit data but will result in a greater loss in accuracy. Furthermore, it suffers a great loss in the performance of the lightweight CNNs, including the MobilenetV1/V2. In fact, as mentioned in [39], most DNPUs have not taken the lightweight CNNs into consideration, and all suffer a great performance loss when processing them. Although [39,41] are specifically designed for lightweight CNNs, their performance is relatively low, as shown in the experiment results. Compared with other works, our design shows high performance for various CNNs. Our work maintains a high computing efficiency for the MobileNetV2/V3-L. It shows that the proposed data transmission scheme is also efficient for the depth-wise convolutional layers. The FC layers are contained in all the tested benches, and they cause no significant loss. This shows that the proposed pipeline strategy for the CNNs is effective.

Compared with other works for CNNs, our design utilizes more hardware resources for the ISP tasks and the RNNs tasks. However, our work still achieves a high-power efficiency measured by the GOPS/W. This shows that the hardware sharing scheme in our architecture is effective, and most hardware resources on the proposed VPU can be applied for the CNNs. This can significantly improve the power efficiency of the VPU because the CNNs are the dominant parts in all the vision tasks.

(2) The Experiment Results of the Hybrid DNNs and the Analysis

Secondly, the hybrid DNNs consisting of the CNNs and RNNs were tested on the proposed VPU and compared with other works. We tested the RNNs alone on the row processor to validate the computing flow for the RNNs. The results show that the runtimes of the LSTM 1000 and GRU 1000 were 5.426 ms and 3.82 ms, respectively.

For the hybrid DNN testbenches, the GOPS/DSP/f and the GOPS/W are also used to evaluate the performance and efficiency of the NPU. Two variations of the LRCN [11] were used as the testbenches. The test results are shown in Table 5.

As shown in Table 5, the hybrid DNNs added negligible delay in the runtime compared with the CNNs. This shows that the proposed pipeline strategy is effective, and the CNNs can mask the processing of the RNNs. Compared with other works, our design shows the highest GOPS/DSP/f. It means that our architecture can achieve better computing performance with the same implementation. This is thanks to the sharing scheme and the reasonable allocation principle for the hardware resources in our work. The pipeline strategy also improves the DSPs’ utilization efficiency, which can run the convolutional layers, the FC layers, and the RNNs concurrently. Besides, although our design increases the amount of the ALUs for the ISP units, it still shows a relatively high-power efficiency. It should be noted that work [27] is implemented with the optimized Deephi Aristotle and Descartes RTL commercial IPs.

#### 5.3.3. The Test of the Complete Vision Tasks

The complete vision tasks consisting of various ISP algorithms and DNNs were executed in our design to test the general performance of the VPU. The results are shown in Table 6. The sum of the runtimes for the ISP algorithms and the DNNs is listed. It is calculated by adding the separate runtime of each ISP algorithm and the DNN together. It was compared with the runtimes of the complete vision tasks.

As shown in Table 6, the runtime of the complete vision task was less than the total runtime of each subtask. The runtime of the complete vision task was very close to the runtime of the DNN. This shows that the pipeline strategy we proposed for the complete vision tasks is effective. The processing of the DNNs masks the execution of the ISP algorithms. Although several ISP algorithms are contained, the power dissipation of the complete vision task was barely larger than that of the DNNs. This is because the DNNs multiplex all the hardware resources for the ISP subtasks, and the ISP subtasks require much less computation than the DNNs. The experiment results indicate that our architecture can efficiently process the vision tasks composed of the ISP and DNNs. It shows negligible overhead in both the hardware resources and the power consumption than the DNPUs.

### 5.4. The Discussion

Our architecture integrates the ISP unit and the DNPU into one VPU. It eliminates the ISP device and the data transmission for the ISP in the vision systems. The experiment results show that this integration has negligible overhead in hardware utilization and power consumption compared with other DNPUs. This is because other DNPUs also contain the units for the non-Mac layers of the CNNs, such as the activating and pooling units, while the ISP tasks and non-Mac layers multiplex the ALU array in our design. Furthermore, the large on-chip memory in the traditional DNPUs is also divided into many Mmems shared for both the ISP and DNNs.

The experiment results also show that the pipelined workflow is efficient for the complete vision task. The ISP algorithms can be executed during the processing of the DNNs, while the convolutional layers can mask the processing of the FC layers and the RNNs. A batch of only three images is required for the complete vision tasks, and the DNNs can be performed on only two images. The pipelined workflow can execute the vision tasks seamlessly, with the high utilization efficiency of the hardware resources.

It should be noted that the convolutional layers account for the most computation of the vision tasks. They also consume the most cycles in the runtime. Therefore, most of the hardware resources of our VPU were allocated to the convolutional layers. Different amounts of the FC layers and RNNs are used in the various vision tasks, and this can result in the idle cycles of the row processor. However, this problem did not cause a significant loss in the general utilization efficiency of the total MACs because the row processor is much smaller than the PE array. For example, in our implementation, the general utilization efficiency of the total DSPs was 92.45%, even if no FC layers/RNNs were contained in the vision tasks, and the row processor stayed idle the whole time. This partly explains why our architecture achieved the high GOPS/DSP/f. Another reason is that our design is applicable for all kinds of CNNs with various kernel sizes, strides, and numbers of input/output channels.

Although our VPU achieved a power efficiency higher than most of the compared DNPUs, its power dissipation was not the lowest. There are two reasons for this. The first one is that our VPU contained more ALUs to process the ISP subtasks. The more important reason is that our VPU utilized fewer BRAMs as the on-chip buffers than other works. When processing the DNNs with more on-chip buffers, the DNPU will generate less data exchange with the external memory. The data transmission and replacement between the on-chip buffers will also be lessened. This will reduce power consumption. However, when implementing the DNPU on the ASIC, it is not practical to equip such big on-chip buffers. As shown in the ASIC-based DNPUs, usually only 200 to 500 Bytes of SRAM is utilized as the on-chip buffer [3,8,15,30]. Therefore, in this paper, we implement the proposed architecture with only 104 BRAMs.

## 6. Conclusions

In this paper, a heterogeneous architecture for the VPU is proposed. It can process the ISP, CNN, and hybrid DNNs simultaneously on one unit. The subtasks can be processed concurrently, and a pipelined workflow is applied to the vision tasks. As a result, the VPU can process the vision tasks seamlessly without any notable waste of the processing modules. It achieves an average performance of 160 GOPS for the DNNs. Moreover, it maintains high performance for all kinds of DNNs and consumes less power than most other works. It can be used in DNN-based vision applications [13,16,17], especially if it is directly applied for the computer vision tasks in the early works of autonomous systems [1,4,48]. There also exist some limitations in this design. For example, processing complicated ISP algorithms on it may produce too long latency, which cannot be masked in the processing of some lightweight DNNs. However, this case rarely happens in practical vision applications. In the future, the VPU can be used to process more machine learning methods with minor modifications. For example, the convolutional layers, FC layers, and RNNs are also used in attention-based models. The proposed VPU can process those models with the aid of a microprocessor. This design can also be applied for other vision systems, including sonar, infrared, terahertz, X-ray, and remote sensing imaging systems.

## Figures and Tables

**Table 1:** The FPGA resource utilization of this work and the comparison with the previous works.

Ref	Year	Device	LUT	FF	BRAM	DSP	DNN ^1^
[37]	2019	Zynq 7100	229,000	107K	386	128	CNN
[38]	2020	XC7K325T	94,763	150,848	165	516	CNN
[28]	2018	XC7Z020	29,867	35,489	85.5	190	CNN
[39]	2020	XC7K325T	173,522	241,175	193.5	704	CNN
[40]	2021	XC7VX690T	278,548	324,033	912	3072	CNN
[41]	2018	Arria 10	163,506	/	24.5Mb	1278	CNN
[26]	2020	XC7Z020	9474	9379	72	/	HDNN
[27]	2018	ZU5EG	117,120	234,240	884	1248	HDNN
[42]	2019	Arria 10	/	/	/	/	HDNN
[9]	2017	XC7VX690T	316,250	321,165	1508	3130	HDNN
[43]	2021	Vertex7	53,078	29,869	465	388	HDNN
This work	2021	XC7K325T	152,264	88,742	104	212	HDNN

^1^ This item denotes the kinds of DNNs supported in each work, and the HDNN is short for the hybrid DNN.

**Table 2:** The Top-1 accuracy of the CNNs with different precisions.

Bit-Width	CNNs			
	**VGG16**	**MobilenetV3L**	**MobilenetV2**	**Densenet121**
Float 32 bit	67.93%	75.2%	72.0%	74.9%
Fixed 8 bit	67.72%	74.1%	71.2%	74.1%

**Table 3:** The experiment results for the ISP tasks and the comparison with other works.

Ref	[2]	[4]	[5]	[1]	[47]	This Work
Senor Resolution	64 × 80	256 × 256	128 × 128	256 × 256	720P	224 × 224
Platform	ASIC	ASIC	ASIC	FPGA	Stratix IV	XC7K325T
PE Array	8 × 10	64 × 64	32 × 128 PE, 32 RP	64 × 64 PE, 8 × 8 PPU	Heterogeneous ^1^	7 × 14
Bit-width for PE	8	1	1 for PE, 8 for RP	1for PE, 16 for PPU	32	8
Freq (MHz)	20	50	100	50	133	200
GOPS ^2^	1.6	12	44	31	37 ^3^	19.6 ^4^
Runtime of						
Demosaic	\	\	\	\	\	56us
8 × 8 DCT	380us		98us	\	\	812us
Median Filter	734us @ 3 × 3		55us @ 8 × 8	\	6.94ms @ 5 × 5	1.96ms @ 3 × 3
Power(mW)	36	630	533	\	98.5	1410

^1^ Only a part of the PEs is used for the ISP tasks in this VPU. ^2^ The performance is counted with 8-bit operations. ^3^ This is the performance of all the PEs in this VPU. ^4^ This is the performance of the ALU array only.

**Table 4:** The experiment results for CNNs on the proposed VPU and the comparison with the previous works.

Ref	Freq (MHz)	Tested DNN	Bit-Width	GOPS	GOPS/ DSP/f	GOPS/W	Power (W)	Runtime (ms)	FPS
[37]	60	VGG16	16	17.19	4.476	27.4	0.627	2269	0.44
[38]	200	VGG16	8	354	3.43	21.45	16.5	82.1	12.18
[28]	214	VGG16	8	84.3	2.073	24.1	3.497	364	2.747
[39]	200	MobileNetV1	8	147.84	1.05	17.9	8.259	3.779	264.6
		MobileNetV2		98	0.696	11.5	8.522	3.07	325.7
		MobileNetV3L		84.84	0.602	9.9	8.57	3	332.7
		DenseNet161		176	1.25	52.5	3.352	41.49	24.1
[40]	200	VGG16	Mixed	2746	4.5	\	\	11.2	89.29
		MobileNetV1		1167.3	1.9			0.47	2127
		MobileNetV2		890.88	1.45			0.34	2941
[41]	133	MobileNetV2	16	170.6	2.007	\	\	3.75	266.6
This work	200	VGG16	8	161.73	3.814	23.7	6.824	197.5	5.06
		MobileNetV2		155.37	3.664	21.6	7.192	3.251	307.59
		MobileNetV3L		149.33	3.521	20.1	7.429	3.002	333.06
		DenseNet161		158.14	3.73	23.6	6.7	104.8	9.54

**Table 5:** The experiment results for the hybrid DNNs on the proposed VPU and the comparison with the previous works.

Ref	Freq (MHz)	Tested DNN ^1^	Bit-Width	GOPS	GOPS/ DSP/f	GOPS/W	Power (W)	Runtime (ms)
[26]	50	LeNet + 2 LSTM128 ^1^	8 for CNN 16 for RNN	/	/	/	/	6
[27]	200	AlexNet + 2 LSTM1024	8 for CNN 16 for RNN	690.76	2.767	86.34	8	9
[42]	268	CRNN	8 for CNN 16 for RNN	646				
[9]	100	AlexNet + 1 LSTM 256	16 for input 12 for weight	36.25	0.1158	1.53	23.69	40
[43]	200	1D CNN + LSTM64	8 for CNN 16 for RNN	49.4	0.637	26.7	1.85	8.295
This work	200	VGG16 + 2 LSTM1000	8 for CNN 16 for RNN	162.7	3.84	23.7	6.864	197.51
		DenseNet161 + 2 LSTM1000		159.23	3.753	23.58	6.751	104.84

^1^ This denotes the hybrid DNN consisting of the LeNet and 2 LSTM with 128 hidden units. Other figures in this column also express the formation of the hybrid DNNs.

**Table 6:** The experiment results for the complete vision tasks.

No.	ISP Algorithms	Tested DNN ^1^	Sum of Runtimes (ms)	Runtime (ms)	FPS	Power (W)
1	Demosiac, DCT, Median filter	MobileNetV3L	5.62	3.14	308	8.11
2	Demosiac, DCT, Median filter	VGG16	200.1	197.48	5.1	6.83
3	Demosiac, DCT, Median filter	VGG16 + 2 LSTM1000	198.37	197.51	5.1	6.94
4	Demosiac, DCT, Median filter	DenseNet161 + 2 LSTM1000	104.83	104.87	9.46	7.04

^1^ Each complete vision task is composed of the listed ISP algorithms and a DNN.
